# A study on the influencing factors of older adults dining satisfaction in community senior canteens based on grounded theory

**DOI:** 10.3389/fpubh.2026.1701296

**Published:** 2026-02-17

**Authors:** Yanyan Sun, Jiongling Wu

**Affiliations:** School of Business, Shanghai Normal University Tianhua College, Shanghai, China

**Keywords:** grounded theory, active aging, community senior dining halls, dining satisfaction, emotional governance

## Abstract

**Introduction:**

Against the backdrop of a rapidly aging population, improving the quality of life for older adults has become an urgent issue. As an innovative model of elderly care services, community-based senior dining halls are gradually emerging as important platforms for promoting active aging in China.

**Methods:**

Based on grounded theory, this study adopts senior community canteens in China as a case study and conducts in-depth interviews with 30 older adults who dine there regularly. It systematically explores the key factors influencing dining satisfaction among older adults in community dining settings.

**Results:**

The findings reveal a progressive mechanism of “environmental stimuli personal appraisal behavioral response” in shaping dining satisfaction. Specifically, the physical environment and service interactions serve as foundational drivers of environmental stimuli; cognitive evaluation and emotional response play moderating roles in the personal appraisal stage; and behavioral responses, such as dining frequency and place attachment, ultimately reflect satisfaction levels.

**Discussion:**

The study further proposes four optimization strategies: institutional demand orientation, emotional spatial design, personalized services, and companion-oriented governance. These offer localized policy recommendations for governments and communities to advance active aging.

## 1 Introduction

The rapid demographic aging in China has generated growing policy and scholarly attention to the everyday living conditions of older adults. Among various community-based eldercare initiatives, community canteens (also known as congregate meal programs) have become an important vehicle for promoting healthy aging, social participation, and aging in place. These canteens provide not only affordable meals but also opportunities for social engagement, emotional comfort, and intergenerational connection (1, 2). Their dual role—as nutritional and social infrastructures—makes them a cornerstone of China's evolving “15-min community life circle” policy framework (3).

Before the COVID-19 pandemic, studies such as Beasley et al. (2018) (4) primarily profiled who attended these meal programs and identified motivations related to cost, convenience, and companionship. However, the pandemic fundamentally altered the operational landscape of community dining services. In response to health risks and policy restrictions, many cities introduced flexible formats, including restaurant-based partnerships, meal voucher schemes, take-away or delivery options, and hybrid public–private management models (5, 6). These flexible formats have redefined the boundaries of community canteens, transforming them from fixed physical venues into adaptive service systems capable of accommodating diverse needs and risk contexts.

In this paper, the term “flexible formats” refers to service arrangements that differ from traditional on-site dining by incorporating three dimensions of adaptation: (1) modal diversity—offering multiple modes such as dine-in, take-away, and voucher-based meals; (2) channel innovation—integrating telephone, online, or app-based ordering and home delivery; and (3) governance hybridity—combining public subsidies with social enterprise or restaurant partnerships. These new formats not only modify the stimuli that older adults experience during dining (e.g., physical environment, social interaction, service process) but also reshape their emotional and cognitive responses, thereby influencing overall satisfaction and intention to revisit.

Meanwhile, a growing body of empirical evidence underscores the importance of commensality—the act of eating together—in promoting psychosocial well-being among older adults. Communal dining enhances feelings of belonging and purpose, mitigates loneliness, and contributes to healthier eating habits (7, 8). The physical and social environment of a canteen thus plays a crucial role not merely as a site of food provision but as a social space where emotional connections and shared identities are cultivated. However, how these environmental and social stimuli are translated into satisfaction and behavioral outcomes remains insufficiently understood.

Despite the growing number of evaluation studies on community meal programs, three key research gaps remain:

(1) Existing studies often focus on outcome indicators such as satisfaction or nutrition, but seldom explore the mechanisms through which environmental, social, and service factors jointly influence these outcomes.
(2) While the Stimulus–Organism–Response (S–O–R) framework offers a promising theoretical lens for understanding consumer behavior in hospitality and service settings (9, 10), it has rarely been applied to community-based eldercare contexts, where emotional and social mechanisms play a critical role.
(3) In the Chinese context, few studies have adopted an inductive, data-driven approach to model the underlying dynamics of senior dining satisfaction, particularly under the new flexible service formats that emerged after COVID-19.

To address these gaps, this study employs grounded theory to construct an integrated mechanism model explaining how environmental, service, and social interaction factors influence the dining satisfaction of older adults in community canteens. Drawing on in-depth interviews and constant comparative analysis, this research explores the stimuli–response pathways that shape older adults' dining experiences and identifies key emotional mediators such as comfort, belonging, and respect. Specifically, the study seeks to answer three research questions: (RQ1) What environmental, service, and social stimuli influence older adults diners' cognitive and emotional appraisals of community canteens? (RQ2) Through which cognitive and emotional mechanisms do these stimuli affect behavioral outcomes such as satisfaction, revisit intention, and advocacy? (RQ3) How can the resulting theoretical model inform service optimization and policy design for age-friendly community dining programs?

By systematically uncovering the mechanisms linking environmental, service, and emotional factors, this study contributes to the literature on healthy aging and community service innovation. Theoretically, it extends the S–O–R framework to the field of eldercare dining, and practically, it provides evidence-based recommendations for enhancing the accessibility, inclusiveness, and emotional quality of community canteens in China's post-pandemic era.

## 2 Literature review

### 2.1 Community dining and older adults' well-being

Community meal programs, also referred to as community canteens or congregate dining services, have long been recognized as vital components of community-based eldercare. These programs provide older adults with not only affordable nutrition but also crucial opportunities for social participation, routine building, and emotional support (1, 2). Empirical studies have shown that regular participation in communal dining contributes to improved dietary quality, enhanced self-rated health, and lower levels of loneliness (6, 7).

In the Chinese context, the establishment of community canteens has been promoted under policies such as the “15-min community life circle,” which aims to ensure access to essential services within walking distance for older citizens (3). Yet, the effectiveness of these canteens depends not only on meal provision but also on the quality of the dining experience—including environment, service, and interpersonal atmosphere—that shapes older adults' sense of dignity and belonging. Consequently, understanding how these factors interact to produce dining satisfaction has become an increasingly important issue in the discourse of healthy aging and aging in place.

### 2.2 Service environment and dining satisfaction

The physical and service environment—or *servicescape*—is a foundational determinant of user satisfaction in public dining settings (9). Previous studies in hospitality and healthcare contexts have demonstrated that spatial layout, lighting, seating comfort, and cleanliness significantly influence perceived quality and emotional comfort (11, 12). In the context of senior dining, accessible design and safety features are particularly critical; when older adults perceive an environment as physically safe and comfortable, they are more likely to form positive emotional responses that foster repeat visits (13).

In addition to tangible conditions, service interactions—including staff politeness, empathy, and personalization—play a decisive role in shaping satisfaction and trust (10, 14). For many older adults, especially those living alone, interactions with canteen staff or peers can provide important sources of emotional affirmation and social connection. Nevertheless, prior research has often treated these service and environmental dimensions independently, neglecting how they interact as a holistic experiential system. This gap calls for integrative models that capture both the material and social stimuli in community dining settings.

### 2.3 Emotional and social mechanisms in community dining

Beyond physical and service attributes, dining satisfaction among older adults is deeply shaped by emotional and social mechanisms. The concept of commensality—the social act of eating together—has been widely linked to psychological benefits such as belonging, identity affirmation, and emotional security (8, 15). When communal meals are infused with care and routine, they can mitigate loneliness and enhance life satisfaction, functioning as an informal social support network (7).

In the Chinese context, where filial norms and family-centered values are deeply rooted, community canteens may also serve as emotional compensation for declining family dining opportunities. Through friendly service interactions and peer companionship, older adults may experience symbolic restoration of family roles and intergenerational bonds (16). Despite this potential, few studies have conceptualized emotional compensation as a mediating mechanism that connects service experiences with loyalty or behavioral intention. Most existing studies remain descriptive, lacking theoretical synthesis or process-level explanation.

### 2.4 Application of the S–O–R framework to eldercare dining

The Stimulus–Organism–Response (S–O–R) framework (17, 18) has been widely applied to examine the psychological mechanisms through which environmental cues influence behavior. In this model, *stimuli* (S) refer to environmental and social factors; *organism* (O) encompasses internal cognitive and emotional processes; and *response* (R) represents behavioral outcomes such as satisfaction, loyalty, or word-of-mouth. Recent hospitality studies have extended this framework to foodservice experiences, showing that sensory design and service quality affect emotional states, which in turn shape revisit intention (6, 12).

However, the S–O–R framework has been underutilized in community-based eldercare contexts. Existing applications primarily address consumer experiences in commercial restaurants or leisure settings, where enjoyment and hedonic value dominate. In contrast, dining among older adults involves functional needs (safety, nutrition), emotional needs (belonging, dignity), and social meanings (community identity). Therefore, applying and extending the S–O–R framework to the context of community canteens can help uncover the multi-layered pathways through which environmental and social stimuli shape both cognitive appraisal and emotional well-being.

Furthermore, the post-COVID-19 shift toward flexible service formats introduces new stimuli (e.g., hybrid delivery, voucher-based dining, digital ordering) that may alter the traditional S–O–R relationships. Investigating these mechanisms empirically can therefore advance both theory and practice.

### 2.5 Summary of research gaps

A critical review of the above literature reveals several gaps. (1) Existing studies focus predominantly on outcome variables (e.g., satisfaction, nutrition) rather than the process mechanisms linking environment, service, emotion, and behavior. (2) The emotional and social dynamics of communal dining—especially the construct of emotional compensation—remain conceptually underdeveloped and empirically untested. (3) The S–O–R framework has not yet been adapted to the eldercare dining context, where physical safety, social connectedness, and emotional well-being coalesce. (4) There is a lack of inductive, data-driven research that reflects the lived experiences of older adults in the rapidly evolving, post-pandemic community dining environment.

These gaps justify an exploratory qualitative study that adopts grounded theory to inductively build a mechanism model explaining how environmental, service, and social stimuli affect dining satisfaction among older adults. Such an approach not only enriches theoretical understanding but also provides practical insights for optimizing the design and management of age-friendly community dining services in China.

## 3 Research design

### 3.1 Research method

Grounded theory, developed by Glaser and Strauss in 1967, is a qualitative research methodology that inductively constructs theory from empirical data. Thus, it is appropriate in research contexts in which theory is lacking or where variables are ambiguous (19). Another important principle of grounded theory is that the researcher does not develop hypotheses in advance; instead, the theory takes shape from first-hand observation and data.

The present study applies the procedural grounded theory method, drawing on the procedures of Xu and Chen (2025) (20). Qualitative data were collected primarily using NVivo 14 qualitative analysis software and a three-level coding method: open coding, axial coding, and selective coding. It intends to reveal, by iterative comparison and inductive analysis, the structural pathway and theoretical framework underlying restaurant dining satisfaction among the older adults.

### 3.2 Research subjects

This study employed a theoretical sampling strategy consistent with grounded theory methodology, emphasizing a dynamic and concept-driven rather than a fixed sampling design. The research was conducted at a community-based senior canteen located in a mature residential district of Shanghai, one of the earliest pilot areas for the municipal *Senior Dining Program*. The site serves approximately 230–260 meals per day and operates under a public–private partnership model: the neighborhood committee provides the venue and oversight, while a contracted social enterprise manages procurement, menu planning, and staffing. This hybrid governance structure—balancing social welfare objectives with cost recovery—represents the mainstream operational logic of urban community canteens in China.

The canteen offers on-site dining, take-away, and limited home-delivery services, catering to older adults with diverse mobility and health conditions. Its clientele—primarily older adults living alone, older couples, and low-income pensioners—closely mirrors the target demographic of Shanghai's municipal senior meal initiatives. As such, the site embodies the typical characteristics and operational challenges of China's community dining programs: ensuring affordability without compromising service quality, balancing efficiency with emotional care, and addressing loneliness alongside nutritional support.

The rationale for site selection lies in its dual significance of typicality and innovation. On one hand, its organizational structure and user profile typify the standard model of municipal community canteens, providing a robust foundation for analytical generalization. On the other hand, the site introduces contextual innovations, such as a volunteer-supported “dialect companion” service that facilitates social connection for isolated older population, and a digitalized meal-tracking system that enhances dietary safety. These features enrich the site's explanatory potential for exploring how environmental and emotional mechanisms jointly shape older adults' dining satisfaction.

A total of 30 older adults were recruited through purposive and snowball sampling, leveraging neighborhood contacts and family referrals. Recruitment followed the principle of theoretical sampling: after the initial round of interviews, emerging categories such as *service attentiveness, spatial comfort*, and *social bonding* guided the inclusion of new participants with differing dining frequencies, living arrangements, and levels of emotional dependence on the canteen. New interviews were purposefully conducted to test and refine conceptual categories until no new variations emerged, indicating theoretical saturation through continuous comparison.

All participants met the following inclusion criteria: aged 65 years or older, possessing normal cognitive and communication abilities, and having dined at the canteen at least three times per week for more than 6 months. Each participant was informed of the study's objectives, provided consent, and was assured confidentiality. The 30 respondents were coded A1–A30; among them, 22 interviews (A1–A22) were used for substantive coding and model development, while eight (A23–A30) were reserved to test theoretical saturation (see Table 1). This approach ensured that the dataset was both empirically representative and conceptually robust, fulfilling grounded theory's emphasis on theoretical adequacy and contextual depth.

**Table 1:** Basic information of research participants.

**ID**	**Gender**	**Age**	**ID**	**Gender**	**Age**	**ID**	**Gender**	**Age**
**A1**	Male	66	A11	Female	65	A21	Female	73
**A2**	Female	65	A12	Female	69	A22	Male	67
**A3**	Female	68	A13	Female	74	A23	Male	80
**A4**	Female	72	A14	Male	76	A24	Male	66
**A5**	Male	71	A15	Female	77	A25	Female	79
**A6**	Male	69	A16	Male	68	A26	Female	69
**A7**	Female	65	A17	Male	84	A27	Male	77
**A8**	Male	80	A18	Male	82	A28	Female	68
**A9**	Male	76	A19	Male	83	A29	Male	65
**A10**	Female	73	A20	Female	69	A30	Female	72

In addition to age and gender, participants varied in household type (living alone, with spouse, or with family), income level (low-income subsidies, basic pension, or self-supported), and health status (self-reported mobility and chronic illness). These background variables were recorded to ensure maximum variation sampling, thereby capturing heterogeneity in living situations and service needs.

Note: The summarized demographic information is now provided in Supplementary Table A1, which presents the distribution of household composition, income level, and self-rated health.

This study adopts a single-case sampling design, focusing on one well-established community canteen that typifies the operation model of municipal senior dining programs in urban China. While this in-depth single-site approach allows for detailed contextual understanding and rich category generation, it also sets clear boundaries for extrapolation. The findings should therefore be interpreted as analytically generalizable—illuminating conceptual mechanisms rather than representing statistical generalization to all canteens or older populations.

Each interview lasted approximately 12–15 mins, consistent with qualitative research protocols for older adults where cognitive load and conversational fatigue must be minimized. Although the interview duration limited depth in some cases, theoretical saturation was achieved through iterative comparison and targeted follow-up interviews, ensuring that the emergent concepts were sufficiently elaborated.

### 3.3 Data collection

Semi-structured, in-depth interviews were conducted to collect data for this study. After reviewing the relevant literature and accumulating fieldwork experience, an interview guide was developed. Initially, a pilot interview was conducted with the help of three participants, and the interview guide was adjusted based on the learning derived from these interviews.

Interviews were conducted using this guide to enable participants to have the space to reflect without losing sight of the issues that were being examined. The objective was to open an interactive conversation in which participants' views could be expressed.

Data were collected through home visits and telephone interviews, each lasting approximately 12–15 min. All interviews were recorded on tape with prior consent from the participants. In total, 486 min of audio data were collected.

As all the participants responded in regional dialects, the researcher transcribed the recordings and converted them into standard Mandarin. After transcription, approximately 30,000 words of usable textual data were obtained. This corpus was subjected to a detailed qualitative analysis to derive concepts and categories.

### 3.4 Reliability and translation validation

To ensure semantic equivalence and minimize interpretive bias during the translation of interview data from regional dialects to Mandarin, a dual-transcription and cross-checking procedure was implemented. The first researcher (native to the local area) transcribed all recordings verbatim and produced the Mandarin version. A second researcher independently reviewed and rechecked the transcripts for lexical accuracy and contextual meaning. Divergent interpretations—particularly idiomatic expressions or emotion-laden phrases—were discussed until full agreement was reached. Analytical memos were used to document these decisions, thereby enhancing reflexivity and transparency.

In addition, during coding, recurring themes and representative quotes were reverified with participants through member checks to confirm that the translated meanings accurately reflected their original intentions.

To enhance transparency and replicability, the semi-structured interview guide used in this study (including core questions, follow-up prompts, and demographic inquiries) is provided as Supplementary Material A. This guide focused on physical space experience, interpersonal interaction, perceived value, and emotional response, consistent with the grounded theory approach to uncovering meaning rather than testing predefined hypotheses.

## 4 Coding and analysis process

### 4.1 Open coding

Open coding is where the interview data were first analyzed. In this stage, the raw transcript underwent line-by-line coding for concept and category recognition. He referred to every sentence and phrase to locate something meaningful and applied constant comparison for refinement.

During open coding, 180 meaning units were extracted, grouped, and refined through iterative comparison. For instance:

- Original statement: “*The chair has a high back, so it doesn't hurt my back” (A12)* → Code: *seat comfort* → Concept: *elder-friendly furniture design*.
- Original statement: “*On rainy days, staff help us with umbrellas” (A22)* → Code: *informal service support* → Concept: *service attentiveness*. These initial concepts were then compared and integrated into seven axial categories. The process evolved iteratively: “environmental stimuli” emerged from physical environment codes, “personal appraisal” from cognitive and emotional responses, and “behavioral response” from frequency and attachment codes. Table 2 visualizes this evolution from data to theoretical model.

**Table 2:** Sample results of open coding.

**Example statement**	**Label**	**Initial concept**	**Initial Category**
A12: “The chair has a high back, so it doesn't hurt my back when I sit.”	Seat comfort	Elder-friendly furniture design	Physical Environment Characteristics
A6: “There are anti-slip mats at the entrance and near the food counter, I'm not afraid of falling.”	Anti-slip flooring	Safety facilities	
A2: “In summer the AC is strong, so I don't sweat while eating; same in winter—it's warm and cozy.”	Temperature control	Environmental comfort	
A21: “The kitchen waste must be cleaned up quickly. There's no odor at the entrance—it's nice.”	Waste disposal efficiency	Hygiene and cleanliness	
A1: “Chef X remembers I don't eat spicy food and reminds me each time if a dish has chili.”	Personalized memory	Staff attentiveness	Service Interaction Attributes
A22: “On rainy days, staff help us collect umbrellas at the door—that's very thoughtful.”	Extra assistance	Informal service support	
A16: “Two meat dishes and one veggie only cost 15 yuan. It's cheaper than cooking myself and saves me from grocery shopping.”	Cost comparison	Functional value (economic)	Cognitive Evaluation
A11: “I often invite my neighbors to come eat and chat together—it's fun.”	Regular social partners	Social value (relational bonding)	
A3: “On regular days there are many dishes to choose from—it feels like home cooking.”	Taste suitability	Functional value (physiological)	
A18: “The food here is good. I told my son not to worry and just focus on his work.”	Family stress relief	Positive emotion (peace of mind)	Emotional Response
A20: “After eating, we can sit and chat on the benches outside the community center.”	Complete supporting facilities	Positive emotion (comfort)	
A8: “When it's crowded, it gets noisy, so I just eat quickly and leave.”	Discomfort from crowding	Negative emotion (environmental stress)	
A9: “I come here almost every day now. I don't even cook at home anymore.”	Dining dependence	Behavioral habit change	Immediate Behavior
A17: “If I moved to my daughter's place, I wouldn't have peers to eat and chat with—I'd miss that.”	Emotional attachment	Place attachment	Long-Term Behavioral Intention
A10: “For us seniors, the price is very reasonable. I often bring my niece here to eat with me.”	Active promotion		

### 4.2 Axial coding

Axial coding is the central part of the grounded theory process. According to Corbin and Strauss, this step involves the reintegration of various dimensions of the categories discovered in the previous open coding step by relating categories to their subcategories in terms of the conditions that give rise to them, their contexts, intervening conditions, action/interactional strategies, and consequences, thus allowing a much more integrated theoretical explanation to emerge.

In this deepening process of establishing more profound links between the delineated categories, the researcher undertook comparative analyses of all the identified categories. Therefore, a logical pattern was revealed, summarized, and systematized into the structure outlined below. This resulted in the relational structure outlined in Table 3.

**Table 3:** Core categories, subcategories, and their thematic meanings from axial coding.

**Thematic meaning**	**Subcategory**	**Core category**
Reasonable seat spacing; clear functional zoning	Spatial layout	Physical environment design
Safety protection facilities; completeness of assistive devices	Age-friendly facilities	
Personalized care; cultural compatibility	Staff service	Service interaction attributes
Convenient procedures; emergency adaptability	Meal service management	
Economic cost-efficiency; health suitability	Functional value	Cognitive evaluation
Family-like experience; perceived dignity preservation	Emotional value	
Sense of belonging; strengthened sense of safety	Positive emotions	Emotional response
Anxiety from crowding; fewer dishes with fewer people	Negative emotions	
Increased frequency; expanded social interaction	Dining behavior adjustment	Short-Term behavior
Place attachment; dish satisfaction; conditional loyalty	Continued usage intention	Long-Term intention

### 4.3 Selective coding

Selective coding, according to Glaser and Strauss, is the final step of grounded theory analysis. It involves the integration of categories generated from axial coding, identification of the core category, and further development of logical relationships between the core category and other categories into an abstract substantive theoretical model. This is the storyline process in which a structured theoretical framework centering on the core category is synthesized^.^

The present study also reveals the mechanism involved when community-based senior canteens provide dining satisfaction for older adults who go through the “Environmental Stimuli—Personal Evaluation—Behavioral Response” process.

**Figure 1:**
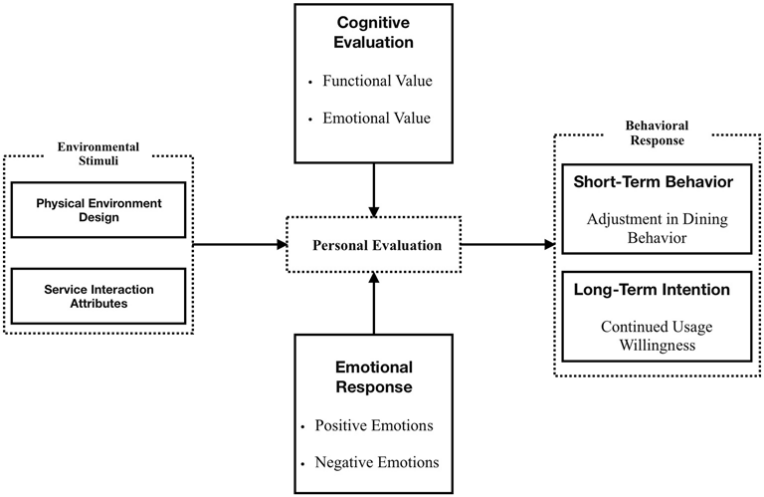
Model of factors influencing dining satisfaction among older adults in community canteens.

In other words, when older adults dine in senior canteens, they first respond to environmental stimuli, such as furniture layout and lighting, and service stimuli, which shape their initial perception of the overall dining environment. This is followed by more subtle factors, such as the cognitive evaluation of food quality and the emotional value of the microenvironment, in forming a personal evaluation. These evaluations shape behavioral responses, such as dining frequency, repeat patronage, and word-of-mouth recommendations. This is shown in Figure 1.

During selective coding, the 15 concepts and 7 categories identified through open and axial coding were integrated into a coherent theoretical model. The process involved comparing and merging categories through constant comparison and theoretical memoing, tracing how “environmental stimuli” (physical and service factors) triggered “personal appraisal” (cognitive and emotional evaluations), which subsequently led to “behavioral responses” (revisit intentions and loyalty). Each category was examined for causal, contextual, and consequential relationships following Strauss and Corbin's paradigm model, culminating in the final “Environmental Stimuli—Personal Appraisal—Behavioral Response” framework (Figure 1).

### 4.4 Theoretical saturation test

Theoretical saturation testing is a relevant step in confirming the validity of the theoretical model developed in this study. Following the 3-level coding procedure discussed earlier, the researcher coded the eight interview transcripts that had been saved in advance. No new concepts or categories have been identified thus far.

For saturation testing, transcripts A25 and A28 were newly coded. Example 1: “*Sometimes I sit near the window for the sunshine—it feels like my old home.” (A25)* → Code: *environmental familiarity* → Category: *spatial comfort*. Example 2: “*The young cook always asks if I'm satisfied—he treats me like his grandma.” (A28)* → Code: *intergenerational empathy* → Category: *service interaction*. No new categories or relationships emerged beyond the established framework, confirming theoretical saturation.

Therefore, the theoretical model is saturated because the core category and its relationships have been fully developed. Thus, the theoretical model can be considered to be explanatory.

## 5 Model interpretation

The emergent model aligns with the Stimulus–Organism–Response tradition but introduces three context-specific refinements. First, the organism stage is partitioned into two distinct mediator tracks—cognitive appraisal (economic and functional evaluation) and emotional response (belonging, dignity, peace of mind)—each with different antecedents and behavioral consequences. Second, we introduce emotional compensation as a construct describing how service attentiveness and peer companionship substitute for absent family ties, thereby strengthening place attachment. Third, we identify boundary conditions: the mechanism is most salient for regular users (≥3 times/week) and those with limited family contact. Together, these refinements confirm and extend prior hospitality and gerontology findings (9, 10, 14), situating the S-O-R framework in an aging-specific servicescape.

### 5.1 Foundational driving role of environmental stimuli

Consistent with servicescape theory (9) and healthcare settings (11), our respondents emphasized safety, seating comfort, and hygiene as prerequisites for dining participation. Unlike leisure hospitality contexts where ambience often plays a primary role, for older adults safety and accessibility function as necessary conditions—without them, emotional engagement is unlikely (13). The physical environment and service interaction (direct antecedents of dining satisfaction) could trigger some primary psychological assessments of older adults via sensory stimuli as a baseline to calibrate needs fulfillment. The physical environment design for the older adults is considered a fundamental guarantee of safety. Reasonable seat spacing and barrier-free paths ensure freedom of movement in public transport. Anti-slip mats and emergency call devices, which are critical safety features for this high-risk age group, directly affect their perception of safety while dining. For example, one respondent explicitly said, “There are anti-slip mats at the entrance and food counters, so I'm not afraid of falling” (A6). Overall, the present study shows that the older adults give positive evaluations of community canteens, and this foundation of positive perception significantly influences their willingness to dine there.

Simultaneously, service interaction attributes stimulate psychological attachment through emotional compensation mechanisms. Personalized caregiving, such as remembering dietary restrictions or speaking in the local dialect, and responsive dining management, such as assistance during extreme weather, jointly constitute a “quasi-family” experience: the chefs modify the dishes to meet the health requirements, and the staff assist in collecting the umbrellas and carrying the bags, making residents feel cared for like family. As respondent A1 and A22 remarked, “Chef X remembers I don't eat spicy food and always tells me which dish has chili in it,” “On rainy days, the staff help us with umbrellas-very considerate.” Such emotional support is highly valued by empty-nest older people, compensating for the absence of family companionship.

This is culturally compatible with service interaction: communication in local dialects promotes trust and eases familiar comfort. “The young ladies here are polite, and their dialect is good-we all understand them well,” said respondent A23.

Therefore, the physical environment and service interaction form a complementary two-driver model, where the former is the foundation for the physical safety mechanism and the latter activates the emotional value mechanism, constructing a complete chain of environmental stimulation that shapes a general positive idea about the dining space.

### 5.2 Facilitative and inhibitory mechanisms of personal evaluation

Thus, the personal evaluation process contains two distinct tracks: cognitive appraisal and emotional response as core mediators between environmental stimuli and behavioral responses.

Earlier foodservice studies emphasize affective mediation (10). Our results refine this picture: cognitive appraisal (economic/functional) predicts short-term utilitarian decisions (e.g., choosing to come that day), while emotional response (belonging, dignity) predicts long-term loyalty and advocacy. This two-track mechanism helps reconcile earlier mixed findings on predictors of continuance. Cognitive evaluation involves a double-track approach: economic rationality and emotional compensation. The economic rational track reflects the function of value in the judgment of satisfaction, commonly clear comparative costs between canteen and home-cooked meals. As respondent A16 said: “Two meat dishes and one vegetable cost just 10 yuan-cheaper than cooking myself, and I don't need to go to the market.”

In contrast, emotional compensation, which is based on emotional value, is a leading driver of long-term attachment. For older adults, especially those who use canteen-based socialization to compensate for a lack of familial companionship, this emotional connection enhances return intention.

Echoing qualitative research on commensality (7, 14), we find that staff attentiveness and repeated social rituals act as emotional substitutes for absent family, creating strong place attachment that manifests in daily attendance and referral behavior. This extends the literature by formalizing emotional compensation as a mediator between service interaction and loyalty. Emotional responses play a deep regulatory role. A sense of belonging is a critical mediating variable between service interaction and behavioral intention, bringing personalized care into place attachment. However, negative emotions may swell in this case. Respondent A8 stated, “Sometimes, it gets too noisy when it is crowded. So I eat up quickly and leave.” These two opposing emotional dimensions function as psychological regulators, where positive emotions enhance behavioral engagement and negative emotions may trigger avoidance or withdrawal.

Micro-detail inputs underpin personal assessment; good-quality service and attention induce a facilitating mechanism in which seniors feel secure and settled, while negative details strengthen negative emotions and levers of cognitive aversion, hence forming an inhibitory mechanism in satisfaction formation.

### 5.3 Behavioral response as the final feedback mechanism

Behavioral responses (dining frequency, advocacy) are sensitive to management practices—menu variety, weekend service, and crowding control. These findings underscore that incremental operational changes (e.g., consistent weekend menus, acoustic control) may yield measurable changes in attendance and well-being, aligning with program evaluation literature in community meal services (4, 6).

Whether older adults continue to dine in community canteens is expressed in terms of short-term intention and long-term commitment to behavior, which is reflected in dining frequency.

In the short term, a high frequency of visits would present evidence of affirmation of service quality: “I come here almost every day now. I don't even cook at home anymore” (A9).

In contrast, a sharp drop in dining frequency may be an early indicator of service failure, typically related to meal provision management. As Respondent A11 noted, “There's a decent variety at lunchtime on weekdays, but on weekends and evenings, there's barely anything to eat.”

Long-term intention is reflected in loyalty. it can be emotional: “If I move in with my daughter, I'll miss the joy of eating and chatting with peers” (A17). or active promotion: “The price here is reasonable for us seniors. I often bring my niece to eat with me as well.” (A10).

Based on the analysis, most older adults had more positive than negative feedback on community canteens. Thus, dining services in the community receive generally good evaluations, and most seniors express their willingness to dine regularly, spreading positive peer influences within their age cohort.

The findings of this study corroborate and extend prior research. Consistent with Bitner's (1992) (9) *servicescape* model, the physical environment was found to be a direct antecedent of satisfaction. However, this study expands on previous hospitality research (10) by identifying emotional compensation as a crucial mediating mechanism unique to older adults in communal settings. Moreover, the dual mechanism of safety perception and emotional belonging aligns with recent aging research emphasizing the psychological importance of dignity and relational connectedness (15). Thus, the present grounded model both confirms and enriches the S–O–R framework through an aging-specific lens.

## 6 Research implications and recommendations

The four proposed optimization strategies directly correspond to the theoretical framework identified in this study. “Institutional Reform” addresses the *stimulus stage* by reshaping external policy stimuli affecting dining satisfaction; “Spatial Optimization” enhances *environmental stimuli* that evoke positive emotional appraisals; “Service Enhancement” targets *cognitive evaluation* through personalized and attentive interactions; and “Emotional Governance” strengthens *behavioral response* mechanisms by fostering emotional attachment and sustained participation. Therefore, each strategy is theoretically grounded in the “Environmental Stimuli—Personal Appraisal—Behavioral Response” mechanism revealed by the grounded theory model.

This study logically reflects the latent mechanism of dining satisfaction in senior canteens, which has profound implications for governments, community operators, and related social organizations. Drawing on this study, the following four dimensions of institutional design, spatial optimization, service improvement, and emotional governance are elaborated:

### 6.1 Institutional reform: from “Subsidy-Oriented” to “Demand-Oriented”

Our analysis indicates that economic evaluation is a primary component of cognitive appraisal (e.g., A16's cost comparison). Therefore, institutional reform should preserve affordable baseline meals while enabling differentiated services for personalization. Practically, a layered subsidy model—basic subsidized meal plus optional paid add-ons for festival menus or personalized dishes—balances affordability and personalization. Evaluation metrics should include not only uptake and cost but also referral intention and self-reported dignity/comfort.

The policy design logic could transform from subsidy to demand. Currently, government subsidies mainly keep the price of meals low, thus engendering a cost-control mentality. In many cases, standard fast-food restaurants are subsidized by the government and renamed “senior canteens,” reducing the canteen to a cost center. This ignores the deep psychosocial needs of older adults to feel “seen” and respected.

This activates a need-feedback-incentive loop through the following:

(1) Quarterly Feedback Collection: Semi-structured interviews by community social workers or third-party evaluators in the local dialect elicited dynamic needs concerning food, services, and space. From the perspective of content analysis, themes can be identified and prioritized without a top-down administrative bias.
(2) Layered and Flexible Subsidy System: Subsidies can be systematized by gradually offering discounts to low-income older people and allowing service providers to charge additional fees for personalized services, such as customized meals and dishes for festivals. This establishes a mixed payment model with basic and market-oriented features.
(3) Linking Subsidies to Word-of-Mouth Metrics: Link government subsidies to indicators such as referral intentions, measured by indices such as “willingness to bring friends,” as measures of effectiveness. This shifts the focus from price competition to experience competition.

### 6.2 Spatial optimization: From “Functional Fulfillment” to “Emotional Scenes”

Because seating, layout, and noise influence perceived comfort and safety (A12, A6), small investments in elder-friendly furniture, anti-slip flooring, and acoustic zoning can increase positive emotional response and attendance. Pilot evaluation can use pre/post perceived comfort scores and changes in dining frequency.

Spatial optimization should shift from crude functionalism toward building spaces that affect people's emotions. While the older adults regard the physical environment in terms of security and accessibility, they also see it as an extension of their memory, identity, and dignity. Strategies:

(1) Micro-memory Design: Introduce local nostalgia in the form of old ration coupons or other items displayed near the entrance or in a corner, with the material of the seats in line with that of older times, to create emotional resonance and place attachment.
(2) Adjustable Social Distancing: Movable screens and plants can divide large dining areas into “active zones” and “quiet zones,” allowing socially active personalities and those sensitive to crowds to participate inclusively.
(3) Sensory and Cultural Adaptation: The environment should correspond to the needs of seasons and culture- for example, cotton cushions in winter, hand fans in summer, and familiar local music, such as opera or revolutionary songs. Multisensory care, including temperature, touch, and sound, nurtures dignity in the dining experience.

Thus, physical space is transformed into an emotional scene- a place that carries remembrance, regulates mood, and facilitates social connection.

### 6.3 Service enhancement: From “Standardized” to “Personalized”

Personalized touches (dietary reminders, dialect-speaking volunteers) were strongly associated with emotional compensation (A1, A22). Operationalizing this requires low-cost tools (simple dietary profiles, staff training on local dialects) and volunteer programs to support companionship.

The core service improvement lies in the transition from a standardized service paradigm to a personalized one by continuously acting on small “exceptions” into institutionalized emotional touchpoints.

Suggestions include:

(1) Dialect Volunteer Plan: Volunteer college students or local youth who speak local dialects can reduce the service distance and meet Respondent A23's preference for dialect communication.
(2) Dynamic Dietary Profile System: Health information can be used to label meals individually, for example, low-sodium, sugar-free, soft food; chefs would refer to profiles in making personalized meal plans, from reminders like “no spicy food” to sets, thus meeting multi-layered health needs at controlled marginal costs.
(3) Emergency Service Protocols: Two emergency service channels-hot meals at home and telephone visits-during extreme weather, such as heatwaves and typhoons, extend the service boundary to the home, thus deepening emotional ties and heightening risk perception.

Thus, informal care is formalized in replicable processes that strike a balance between the sustainability of public welfare and individual dignity.

### 6.4 Emotional governance: From “Meal Support” to “Companionship Support”

Interventions that actively create stable small groups or intergenerational activities can strengthen social bonding and retention (A11, A17). Monitoring should include social network indicators (e.g., count of stable dining partners) and retention/referral metrics.

Emotional management thus brings the aim of senior canteens from nutrition supplements to intervening loneliness. According to the findings, social bonding is more predictive than food quality in terms of long-term loyalty. Therefore,

(1) “Dining Table Socialization” Scheme: Community workers match solo diners with nearby similar residents with compatible personalities to establish stable “dining partners” and alleviate social isolation.
(2) Intergenerational Sharing Days take place monthly, during which schoolchildren or youth volunteers eat with the older population. Intersubjective interactions can fill in for missing family support, as Respondent A18 stated, “I tell my son not to worry about me.”
(3) “Canteen+” Hybrid Space: Facilities like “Silver Stage” are where the older adults sing local operas and show their calligraphic works. This will move the canteen from a point of consumption to a point of emotional contact within the neighborhood, thus breaking psychological barriers for first-time users and establishing emotional capital in a self-reinforcing cycle.

### 6.5 Limitations and future research

Although this study is based on a single-site qualitative design, the selected canteen's operational scale, management model, and clientele composition reflect the typical characteristics of urban senior dining programs in Shanghai. Therefore, the findings possess analytical rather than statistical generalizability, offering conceptual insights applicable to similar urban community contexts. Despite its contributions, this study has several limitations. First, because the data derive from a single community canteen, the proposed mechanism model should be viewed as context-dependent. Future multi-site or cross-regional comparative studies could test the model's transferability under different cultural, geographic, and management conditions. Second, while grounded theory offers rich qualitative insights, combining it with quantitative validation methods (e.g., structural equation modeling) could further verify the causal pathways among identified factors. Finally, longitudinal observation of older adults' dining behaviors could deepen understanding of how satisfaction evolves over time in response to environmental and social changes.

## Data Availability

The original contributions presented in the study are included in the article/Supplementary material, further inquiries can be directed to the corresponding author/s.
