# Supplementary material for: A study on the influencing factors of older adults dining satisfaction in community senior canteens based on grounded theory
Source: Front Public Health. 2026 Feb 17;14:1701296. doi: 10.3389/fpubh.2026.1701296 (PMC12954619; doi:10.3389/fpubh.2026.1701296)
Supplement: Supplementary file 1 [file Data_Sheet_1.pdf]

Supplementary Table A1. Demographic Characteristics of Interview Participants (n = 30)

Variable	Category	n	%	Note
**Gender**	Male	13	43.3	
	Female	17	56.7	
**Age group (years)**	65–69	10	33.3	
	70–74	9	30.0	
	75–79	7	23.3	
	≥80	4	13.3	
**Living arrangement**	Living alone	9	30.0	Reflects “empty-nest” situation
	With spouse	12	40.0	
	With children/family	9	30.0	
**Income level**	Low-income subsidy recipient	8	26.7	Based on local social welfare registration
	Basic pension	15	50.0	
	Self-funded	7	23.3	
**Self-rated health**	Good	13	43.3	
	Fair	12	40.0	
	Poor	5	16.7	
**Dining frequency**	3–4 times/week	11	36.7	
	5–6 times/week	9	30.0	
	Daily	10	33.3	
**Duration of participation**	6–12 months	7	23.3	
	1–2 years	12	40.0	
	≥2 years	11	36.7	
