# Supplementary material for: A study on the influencing factors of older adults dining satisfaction in community senior canteens based on grounded theory
Source: Front Public Health. 2026 Feb 17;14:1701296. doi: 10.3389/fpubh.2026.1701296 (PMC12954619; doi:10.3389/fpubh.2026.1701296)
Supplement: Supplementary file 2 [file Data_Sheet_2.pdf]

Interview Guide

A1. Introduction to the Interview

Opening Script:

Thank you very much for taking part in this interview. We are conducting a study on dining experiences in community canteens for older adults.

Your opinions will help us understand how to improve service and space design.

The interview will take about 15 minutes. Your participation is voluntary, and all information will remain confidential.

A2. Interview Structure and Main Questions

Section	Key Question	Probing Prompts / Follow-ups	Analytical Purpose (Coding Link)
**1.Dining Habits and Motivations**	Could you describe how often you come to this canteen and why you choose to dine here?	What makes you prefer this canteen compared with cooking at home or eating elsewhere?	Identify *behavioral motivation* and *functional value*.
**2.Physical Environment Experience**	How do you feel about the canteen's environment (seating, lighting, temperature, layout)?	Do these factors make dining easier or more comfortable for you?	Generate *environmental stimuli* concepts (safety, comfort, accessibility).
**3.Service Interaction**	How would you describe the staff's service attitude or behavior?	Any memorable service experience? Any aspect you would like to see improved?	Identify *service interaction* and *personalized care*.
**4.Emotional Feelings**	How do you feel emotionally when eating here? (e.g., relaxed, cared for, lonely, happy)	Do you think dining here affects your mood or your sense of belonging?	Explore *emotional response* and *psychological satisfaction*.
**5.Social Experience**	Do you interact with other diners? What kinds of conversations or relationships do you have here?	Has this place helped you make friends or feel connected with others?	Extract *social bonding* and *companionship satisfaction*.
**6.Cognitive Evaluation**	Do you think the price, food quality, and service are reasonable?	How would you compare it with eating at home or outside restaurants?	Identify *cognitive appraisal* and *perceived fairness/value*.
**7.Behavioral Intention**	Will you continue dining here in the	Would you recommend this place	Derive *behavioral response* and *loyalty*

	future? Why or why not?	to others?	*intention*.
**8. Suggestions**	What improvements would you suggest for this canteen?	What would make you feel more satisfied or comfortable?	Gather practical insights for *policy recommendations*.

A3. Demographic Questions

1. Age
2. Gender
3. Marital status (living alone / with spouse / with children)
4. Monthly income range
5. Frequency of dining at the canteen (times per week)
6. Duration of using the canteen (months or years)
7. Self-rated health (good / fair / poor)
8. Whether living in the same community as the canteen

A4. Closing Statement

Thank you very much for sharing your experiences.
Is there anything else about your dining experience you'd like to add?
Your input is very valuable and will help improve community services for older adults.
